# Characterization and Comparison of the Two Mitochondrial Genomes in the Genus *Rana*

**DOI:** 10.3390/genes14091786

**Published:** 2023-09-11

**Authors:** Yan-Mei Wang, Chi-Ying Zhang, Si-Te Luo, Guo-Hua Ding, Fen Qiao

**Affiliations:** 1 Laboratory of Amphibian Diversity Investigation, College of Ecology, Lishui University, Lishui 323000, China; wymtina@njfu.edu.cn (Y.-M.W.); guwoding@lsu.edu.cn (G.-H.D.); 2 College of Life and Environmental Sciences, Hangzhou Normal University, Hangzhou 311121, China; chixyzhang@163.com; 3 School of Life Sciences, Xiamen University, Xiamen 361005, China; lstxmu@gmail.com

**Keywords:** mitogenome, Ranidae, *Rana*, gene arrangement, phylogeny

## Abstract

The mitochondrial genome (mitogenome) possesses several invaluable attributes, including limited recombination, maternal inheritance, a fast evolutionary rate, compact size, and relatively conserved gene arrangement, all of which make it particularly useful for applications in phylogenetic reconstruction, population genetics, and evolutionary research. In this study, we aimed to determine the complete mitogenomes of two morphologically similar *Rana* species (*Rana hanluica* and *Rana longicrus*) using next-generation sequencing. The entire circular mitogenome was successfully identified, with a length of 19,395 bp for *R. hanluica* and 17,833 bp for *R. longicrus*. The mitogenomes of both species contained 37 genes, including 13 protein-coding genes (PCGs), two ribosomal RNA genes, 22 transfer RNA genes, and one control region; mitogenome size varied predominantly with the length of the control region. The two synonymous codon usages in 13 PCGs showed that T and A were used more frequently than G and C. The ratios of non-synonymous to synonymous substitutions of all 13 PCGs were <1 in the *Rana* species, indicating that the PCGs were under purifying selection. Finally, phylogenetic relationship analyses suggested that *R. hanluica* and *R. longicrus* were classified in the *R. japonica* group. Our study provides valuable reference material for the taxonomy of the genus *Rana*.

## 1. Introduction

The mitochondrial genome (mitogenome) possesses several valuable characteristics (e.g., limited recombination, maternal inheritance, rapid evolutionary rate, small size, and relatively conserved gene arrangement) for utilization in phylogenetic reconstruction, population genetics, and evolutionary studies [1,2]. Understanding the factors driving mitogenomic evolution may offer crucial insights into these benefits [2]. As anticipated, the mitogenomes of Anura are double-stranded circular molecules with a length of 16–24 kbp [3], encompassing two ribosomal RNA (12S and 16S rRNAs) genes, 22 transfer RNA (tRNA) genes, 13 protein-coding genes (PCGs), and one control region (CR; also known as the D-loop) [1,4,5,6]. The mitogenome is extensively employed in evolutionary studies of diverse species [7], and the evolution of tRNA genes and novel gene orders in the mitogenome can aid in determining phylogenetic relationships [5]. 

True frogs of the genus *Rana* Linnaeus, 1758 (Ranidae) have a wide distribution across Eurasia and the Americas [8]. Owing to their body coloration and habitat preferences, species within this genus are colloquially called brown frogs or wood frogs [9,10]. The taxonomy of *Rana* (brown frogs) has been the subject of intense debate over the past 20 years [8,11]. 

A recent phylogenetic analysis indicated that *Rana sensu lato* may consist of nine clades, namely the genus *Rana* [12]. At present, the well-established genus *Rana* comprises 54 species globally [13], with 26 species documented in over 30 provinces in China [8]. To date, complete mitogenome sequences of only 14 *Rana* species were deposited in GenBank.

In the genus *Rana*, one of the major species groups of Chinese brown frogs is *Rana longicrus*, and the majority of southern Chinese brown frogs fall within this species group [14]. Previous studies have proposed that the Long-legged Brown Frog (*R. longicrus*) should also be classified under this species group, owing to morphological and nucleotide differences [13,15]. However, prior research indicated that *R. longicrus* belongs to the *R. japonica* group [15,16]. Alongside taxonomic revisions, recent investigations on this subgenus have unveiled several new species in China, underscoring the incomplete understanding of the subgenus *Rana* [9,11,17].

The Hanlui Brown Frog (*Rana hanluica*), named after its breeding season, which is around the “Cold Dew Festival”, falling in one of the 24 solar terms of the Chinese lunar calendar, is endemic to China. Its type locality is in Shuangpai, Hunan Province [18]. While most *Rana* species distributed in southern China are part of the *R. japonica* group [17,19], Fei et al. [13] classified six *Rana* species from southern China, including *R. hanluica*, under the *R. longicrus* group. The uncertain systematic studies of the genus *Rana* have hindered a comprehensive understanding of species diversity and biogeographic patterns within the genus [12]. Therefore, further investigations based on complete mitogenome data might enhance our comprehension of the phylogenetic relationships among these Ranidae species [20].

In this study, we successfully determined the complete mitogenome sequences of the two *Rana* species via Illumina sequencing and compared them with those of related species based on mitochondrial structure. We also analyzed the phylogenetic position and relationships of *R. longicrus* and *R. hanluica* by reference to the available complete mitogenomes of the family Ranidae. Our findings offer valuable insights for future research on the evolutionary genetics, species delineation, and phylogeny of the genus *Rana*.

## 2. Materials and Methods

### 2.1. Sample Collection

The specimen of *R. longicrus* (voucher number: LSU20210423MXZY001) was collected from the Fujian Junzifeng National Nature Reserve, Mingxi, Fujian Province, China (E 117.4977°, N 26.3357°), in April 2021. The specimen of *R. hanluica* (voucher number: LSU20201009LSLD001) was collected from the Liandu Fengyuan Nature Reserve, Lishui, Zhejiang Province, China (E 119.8266°, N 28.1934°). Both specimens were deposited in 75% ethanol and stored in the Museum of the Laboratory of Amphibian Diversity Investigation at Lishui University (Lishui, Zhejiang, China). Total genomic DNAs of the two specimens were extracted from the muscle using the DNA easy Tissue Kit (TransGen Biotech Co., Beijing, China). The quantity and quality of extracted DNA samples were determined using a NanoDrop2000 spectrophotometer (Thermo Fisher Scientific, Waltham, MA, USA) and agarose gel electrophoresis, respectively.

### 2.2. Assembly and Annotation

Sequencing libraries of the two *Rana* species were generated using an Illumina Truseq™ DNA Sample Preparation Kit (Illumina, San Diego, CA, USA). The prepared libraries were loaded onto an Illumina HiSeq 6000 platform for paired-end 150 bp sequencing (Novogene Bioinformatics Technology Co., Ltd., Tianjin, China). Complete mitogenomes were assembled de novo based on clean data using the GetOrganelle toolkit [21] and annotated using MitoZ v2.4 [22]. Initial sequencing data were aligned to the mitogenome using Burrows–Wheeler alignment, resulting in two SAM alignment files. The SAM files were subsequently transformed into the BAM format and organized using Samtools to optimize the efficiency of the subsequent image processing steps. Coverage statistics were determined for each position along the mitogenome by analyzing the sorted BAM files of the same depth. The outcome was a text file containing coverage details. To further analyze this information, the coverage files were imported into R and processed using the tidyverse package. Within R, the data were organized according to position, allowing the calculation of the average coverage per position. Notably, the entire circular mitogenomes of the two target species were fully covered by the reference sequence. The reference sequence comprehensively covered the entire circular mitogenome of the two target species, and this thorough coverage resulted in an average read coverage depth of 181.66× in *R. hanluica* and 160.51× in *R. longicrus* (Figure S1). This indicated that the sequencing data possessed high quality and reliability, enabling the provision of more accurate genomic information.

### 2.3. Bioinformatic Analysis

Graphical maps of the *R. longicrus* and *R. hanluica* mitogenomes were produced using the CGView Server (http://cgview.ca/, accessed on 12 December 2021). The base composition and relative synonymous codon usage (RSCU) were obtained using MEGA X [23]. Besides *R. hanluica* and *R. longicrus*, 22 mitogenomes of other 18 species in Ranidae were also collected, including two *Amolops* species, two *Odorrana* species, and 14 *Rana* species (Table S1). The skew in nucleotide composition was calculated by the GC and AT skew, which was measured according to the following formulas [24]: AT skew = (A − T)/(A + T); and GC skew = (G − C)/(G + C). To evaluate the divergence of the paralogous genes, we analyzed synonymous (Ks) and non-synonymous (Ka) substitutions by using DnaSP 5.1 [25]. To evaluate possible evolutionary patterns across the 13 PCGs, the Ka/Ks ratios (namely the ω value) were calculated among the 16 representative *Rana* species. If a selection does not affect fitness, Ka = Ks and ω = 1. If non-synonymous mutations are deleterious, purifying selection will reduce their fixation rate, such that Ka < Ks and ω < 1. If non-synonymous mutations are favored by Darwinian selection, it results in Ka > Ks and ω > 1 [26].

### 2.4. Phylogenetic Analyses

Eighteen previously published *Rana* mitogenomes [27,28,29,30,31,32,33,34,35,36] along with the newly determined sequences of the two species, were used in the phylogenetic analyses to discuss the relationships within the family Ranidae. Four other species (*Amolops ricketti*, *Amolops wuyiensis*, *Odorrana graminea*, and *Odorrana schmackeri*) were used as outgroups [37,38,39]. The genomes were aligned using MAFFT v7.388 [40]. The concatenated set of nucleotide sequences, including 13 PCGs and 2 rRNA genes, was used for phylogenetic analyses, which were performed following the Bayesian inference (BI) and Maximum Likelihood (ML) methods in MrBayes v3.2.7 and IQ-TREE2 v2.1.2, respectively. In the BI analysis, the best-fit substitution model (GTR + G + I) was selected, and the following settings were applied: two simultaneous runs of 10,000,000 generations were conducted for the matrix; the number of Markov chain Monte Carlo generations was set to ten million; the sampling frequency was set to 1000, and the burn-in was set to 1000. The Bayesian runs achieved sufficient convergence when the average standard deviation of the split frequencies was <0.01. ML analyses were performed using the best-fitting nucleotide substitution model (TPM2 + F + I + G4), which was selected by ModelFinder [41,42]. Support for the inferred ML tree was inferred by bootstrapping with 1000 replicates. 

## 3. Results and Discussion

### 3.1. General Features of Mitogenomes of Two Rana Species

**Figure 1:**
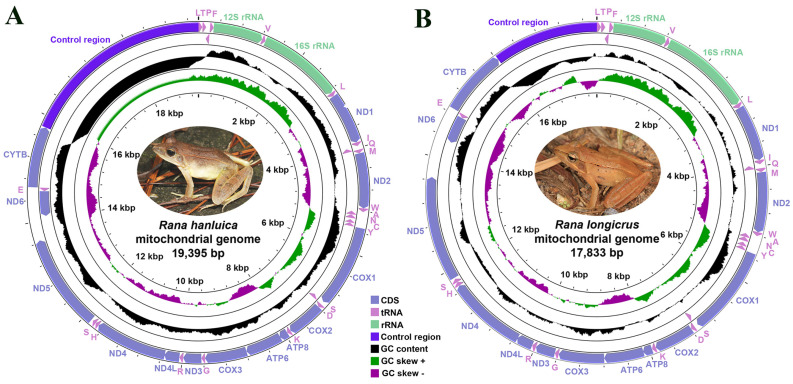
Circular map of the complete mitochondrial genomes of (**A**) *Rana hanluica* and (**B**) *Rana longicrus*.

As in most ranid frogs, the two *Rana* species had circular mitogenomes (Figure 1). The size of the mitogenome was larger in *R. hanluica* (19,395 bp) than in *R. longicrus* (17,833 bp) (Table 1), and both comprised 13 PCGs, two rRNA genes, 22 tRNA genes, and a CR (Table S2), the length of which was inconsistent between the two species (Figure 1). Differences in mitogenome length between the species were predominantly attributed to the overall length of the CR, which in turn differed in both replicates and lengths of various short-repeat sequences within it [43]. The mitochondrial genomes of *R. longicrus* and *R. hanluica* were compact with only a few intergenic spaces and overlapping gene junctions. Furthermore, among the 13 mitochondrial PCGs, only one gene (ND6) was encoded by the L-strand, which is the typical arrangement of 13 PCGs [33]. The remaining 12 genes were encoded by H-strands (Table S2). Other species in the genera *Rana* and *Odorrana* [44,45] also have similar distribution characteristics [33,46], as the arrangement of genes within vertebrate mitogenomes is highly conserved [6,47,48,49]. 

The mitogenomes of *R. longicrus* and *R. hanluica* were biased toward AT nucleotides, ranging from 55.6% (*R. longicrus*) to 59.1% (*R. hanluica*) (Table 1). This A-T-rich pattern reflects typical sequence features of the vertebrate mitochondrial genome [50]. Furthermore, in the two *Rana* mitogenomes, AT-skews and GC-skews were both negative, indicating that the mitogenomes of both species contained a higher percentage of AT vs. GC nucleotides, which is consistent with the lowest frequency of G content in typical amphibian mitogenomes [3,48,51]. Generally, amphibian mitogenomes show a strong bias in nucleotide composition, with third codon positions being the most strongly affected by nucleotide compositional bias and skew [48]. 

### 3.2. Protein-Coding Genes and Codon Usage

A summary of the genes constituting the *R. longicrus* and *R. hanluica* mitogenomes is given in Table S2. The total length of all the PCGs in *R. longicrus* (11,288 bp) exceeded that in *R. hanluica* (11,279 bp). The longest PCG was ND5, and the shortest was ATP8 in the two *Rana* mitogenomes. Although gene rearrangements are frequently observed in amphibians [6], they were not detected in the two *Rana* species. Ten of the thirteen PCGs used the standard ATG as the start codon in the two *Rana* species, except for ND1, COX1, and ND4L, which were initiated by the GTG codon. Although TTG is an uncommon start codon among PCGs, it is often found in amphibians [51]. However, similar results were not found in the two *Rana* species. Four typical stop codons were found, TAG, AGG, AGA, and TAA, and incomplete terminal codons (TA-, T-) were also present in the two *Rana* mitogenomes.

**Figure 2:**
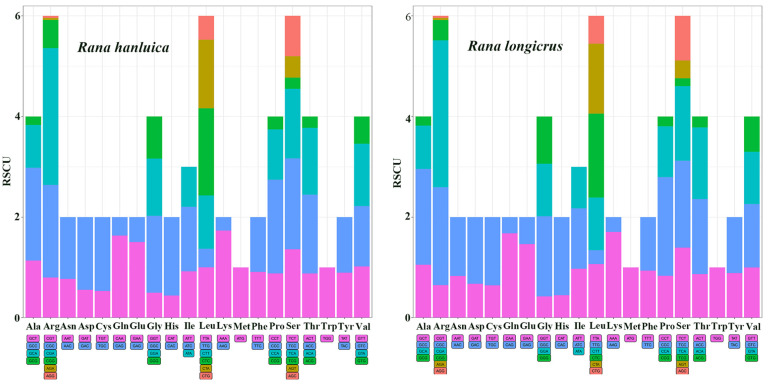
Codon number and relative synonymous codon usage of protein-coding genes in *R. hanluica* and *R. longicrus* mitogenomes.

Subsequently, we evaluated the RSCU in the 13 PCGs to determine their preference for specific synonymous codons, as summarized in Table S3. Our results showed that T and A were more frequent than G and C, indicating a strong A or T bias in the third codon position. The most common amino acids in these two *Rana* mitogenomes were Arg, Leu, and Ser (Figure 2).

**Figure 3:**
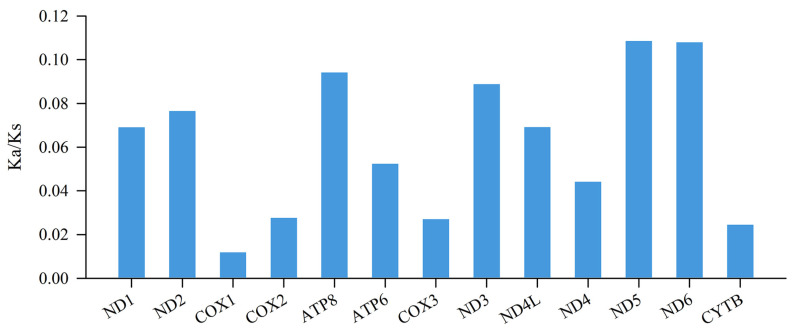
The evolutionary rate indicated by the ratios of non-synonymous to synonymous substitutions (Ka/Ks) for each PCG among the 16 representative *Rana* mitogenomes.

Synonymous and non-synonymous substitutions are important in understanding evolution [52], with selection pressure analysis being an indispensable part of evolutionary analysis. The Ka/Ks ratios of all 13 PCGs were much lower than those in the *Rana* species (Figure 3), indicating that the PCGs were under purifying selection [26]. COX1 had the lowest Ka/Ks ratio (0.012), suggesting that this gene was under the strongest selective pressure. In contrast, ND5 exhibited the highest Ka/Ks ratio (0.108), indicating that it was under the least selective pressure. The ratio varied among the 13 PCGs reflecting the different functional constraints among these genes [53]. In addition, we failed to detect significant adaptive mutations in the 13 mitogenomic PCGs; therefore, purifying selection may be the predominant force governing mitogenome evolution in *Rana* species [54].

### 3.3. Transfer RNA and Ribosomal RNA Genes

Twenty-two tRNA genes were scattered across the two *Rana* mitogenomes: 65 bp for tRNA-Cys and 73 bp for tRNA-Asn. All tRNA genes appeared highly A + T biased; the AT-skew was slightly positive, and the GC-skew was negative in the 22 tRNA genes of the two *Rana* species (Table 1). Among these tRNA genes, 8 (tRNA-Pro, tRNA-Gln, tRNA-Glu, tRNA-Ala, tRNA-Asn, tRNA-Tyr, tRNA-Cys, and tRNA-Ser) were encoded by L-strands, and the other 14 tRNA genes were encoded by H-strands (Table S2). Furthermore, we found that the lengths of all rRNA genes (2509 bp in *R. hanluica* and 2506 bp in *R. longicrus*) were similar to those of other *Rana* species (e.g., 2510 bp in *R. omeimontis*, 2505 bp in *R. kukunoris*, and 2511 bp in *R. pyrenaica*) [30,33,46]. The 12S and 16S rRNA genes are located between tRNA-Phe and tRNA-Leu and are separated by tRNA-Val, which is a common feature in vertebrate mitogenomes [54]. The A + T content of the two rRNA genes was 55.4% in *R. hanluica*, which was lower than that in *R. longicrus* (55.9%). Additionally, they had a positive AT skew and a negative GC skew.

### 3.4. Noncoding Regions

The noncoding regions in the mitogenome of both *Rana* species included the CR and a few intergenic spacers. The CR is located between tRNA-Leu and CYTB, which harbors several conserved blocks for the replication and transcription of the mitogenome. The CR of *R. hanluica* was 3602 bp, which was longer than that of *R. longicrus* (1807 bp) (Table 1). CR contains signals for the initiation and regulation of mitogenomic transcription and replication [55]. Metazoan mitogenomes usually have CRs that vary in length among species [56]. The A + T content (78.8%) of the CR in *R. hanluica* is the highest known to date among *Rana* species. In addition, both *R. hanluica* and *R. longicrus* showed negative AT-skew and GC-skew in the CR.

### 3.5. Phylogenetic Analysis

**Figure 4:**
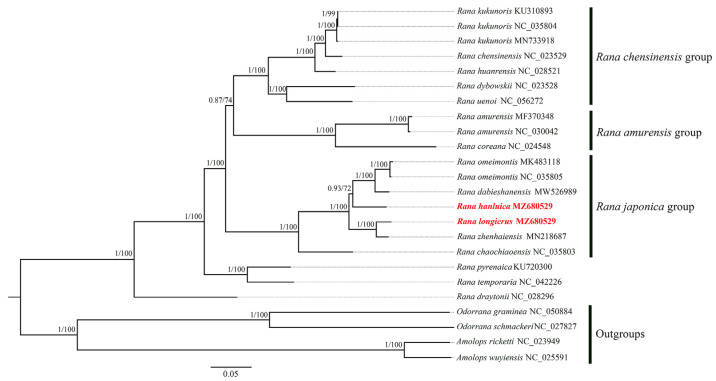
Phylogenetic tree for the genus *Rana* using BI and ML analyses based on nucleotide sequences. Bayesian posterior probability (BPP) and bootstrap values (BP) of each node are shown as BPP/BP, with maxima of 1.00/100. Samples sequenced in the present study are highlighted in red.

The final concatenated 13 PCGs and two rRNA genes for the 22 *Rana* samples were 13,921 bp in size, including 6695 variable sites of which 942 were singleton sites. The two phylogenetic reconstruction methods (ML and BI) yielded identical tree topologies that favored the clades and/or relationships of the family Ranidae (Figure 4). 

The phylogenetic analysis in this study showed that the trees generated via both ML and BI were identical to each other in topological structure (Bayesian posterior probability ≥ 0.87; bootstrap value ≥ 72%), and they both supported the following classification and phylogenetic relationships of the *Rana* species. Three clades corresponded to previously recognized species groups, namely the *R. chensinensis* group, *R. amurensis* group, and *R. japonica* group. The *R. japonica* group was the sister group of a clade comprising the *R. chensinensis* and *R. amurensis* groups. Our results showed that *R. longicrus* and *R. zhenhaiensis* clustered on one branch, *R. hanluica* was close to *R. dabieshanensis* and *R. omeimontis*, and these five *Rana* species were classified in the *R. japonica* group (Figure 4). Other results suggested that *R. pyrenaica* and *R. temporaria* were clustered into a clade, and these, along with *R. draytonii*, were the two main conspicuous subclades (Figure 4), which may not be consistent with the other study [30].

Overall, the reconstructed phylogeny at the genus level in our study verified that *R. longicrus* and *R. hanluica* belong to the *R. japonica* group, which is consistent with the results of Wang et al. [57] and Wan et al. [17]. Further studies are required to clarify the phylogenetic position of the genus *Rana*. Although we did not fully resolve the taxonomic status within the genus *Rana*, this study provides valuable reference material for elucidating the taxonomy of the genus *Rana*. 

## 4. Conclusions

We present the complete mitogenomes of *R. hanluica* and *R. longicrus*, with sizes of 19,395 and 17,833 bp, respectively. The mitogenome structures of the two *Rana* species share many features with most previously reported ranid frogs, including 37 genes in the mitogenomes (13 PCGs, 22 tRNA genes, and two rRNA genes). The phylogenetic tree was analyzed by rebuilding topological trees (ML and BI) with a dataset of 13 PCGs and two rRNA genes, which showed that *R. longicrus* and *R. hanluica* were classified in the *R. japonica* group. Our results provide valuable insights for further studies on the evolutionary genetics, species delimitation, and phylogeny of the genus *Rana*.

## Figures and Tables

**Table 1:** The mitogenome composition of the two *Rana* species.

Region	Size (bp)		A + T Content (%)		G + C Content (%)		AT−Skew		GC−Skew	
	RH	RL	RH	RL	RH	RL	RH	RL	RH	RL
Whole genome	19,395	17,833	59.1	55.6	40.9	44.4	−0.045	−0.007	−0.314	−0.328
PCGs	11,279	11,288	53.9	54	46.1	46	−0.088	−0.082	−0.322	−0.321
rRNA genes	2509	2509	55.4	55.9	44.6	44.1	0.157	0.162	−0.15	−0.148
tRNA genes	1530	1530	57.6	56.9	42.4	43.1	0.015	0.024	0.045	0.032
CR	3602	1930	78.8	64.2	21.2	35.8	−0.134	−0.031	−0.251	−0.294

RH: *Rana hanluica*; RL: *Rana longicrus*.

## Data Availability

The datasets presented in this study can be found in online repositories. The names of the repository/repositories and accession number(s) can be found below: NCBI (accession: MZ680528, MZ680529, SRR15045243, and SRR15045242).

## Supplementary Materials

The following supporting information can be downloaded at https://www.mdpi.com/article/10.3390/genes14091786/s1; Table S1: GenBank accession numbers of the ranid species used in the phylogenetic trees; Table S2: The mitogenome characteristics and location of the two *Rana* species; Table S3: RSCU information for the mitochondrial protein-coding genes of *R. hanluica* and *R. longicrus*; and Figure S1: Mitogenome coverage map of (A) *Rana hanluica* and (B) *Rana longicrus*.
