# Supplementary material for: Characterization and Comparison of the Two Mitochondrial Genomes in the Genus Rana
Source: Genes (Basel). 2023 Sep 11;14(9):1786. doi: 10.3390/genes14091786 (PMC10530863; doi:10.3390/genes14091786)
Supplement: Supplementary file 1 [file genes-14-01786-s001.zip › genes-2564242-supplementary.pdf]

Characterization and comparison of the mitochondrial genomes from two *Rana* species

Yan-Mei Wang, Chi-Ying Zhang, Si-Te Luo, Guo-Hua Ding, Fen Qiao

Supplemental materials

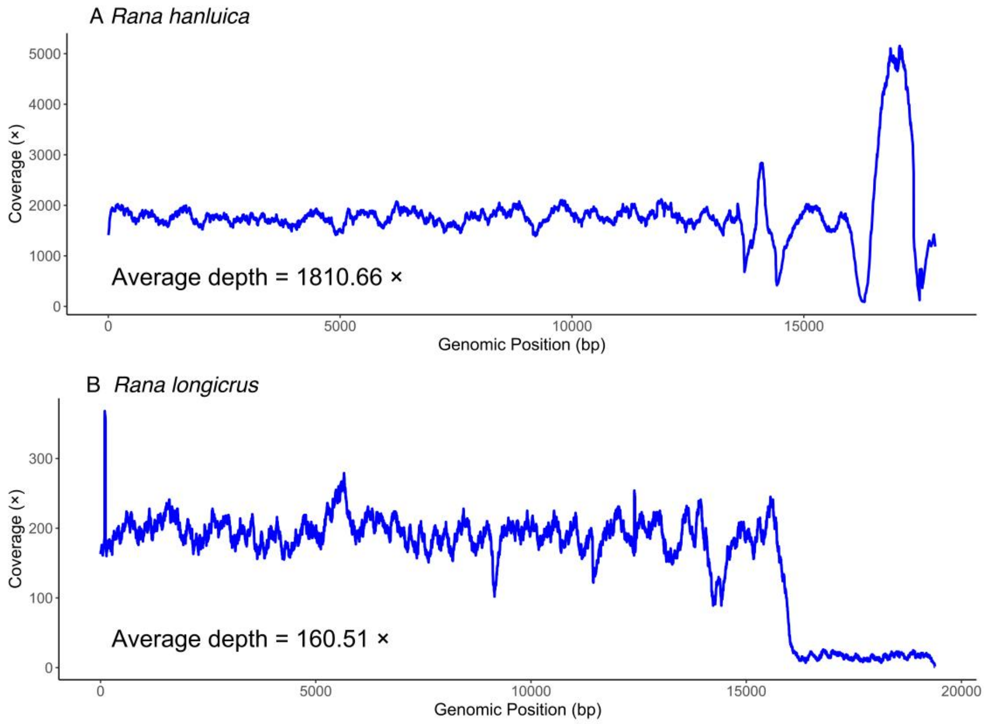

Figure S1. Mitogenome coverage map of (A) *Rana hanluica* and (B) *Rana longicrus*.

Table S1 GenBank accession numbers of the ranid species used in the phylogenetic trees.

ID	Genus	Species	Accession No.	Mitogenome size (bp)	Quality	References
1	*Amolops*	*Amolops ricketti*	NC_023949	17,772	complete	Li et al., 2016c
2		*Amolops wuyiensis*	NC_025591	17,797	complete	Huang et al., 2016
3	*Odorrana*	*Odorrana graminea*	NC_050884	18,106	complete	Jin et al., 2020
4		*Odorrana schmackeri*	NC_027827	18,302	complete	Unpublished
5	*Rana*	*Rana amurensis*	MF370348	20,571	complete	Liu et al., 2017
6		*Rana amurensis*	NC_030042	18470	complete	Unpublished
7		*Rana chaochiaoensis*	NC_035803	18,591	complete	Unpublished
8		*Rana chensinensis*	NC_023529	18,808	complete	Li et al., 2016a
9		*Rana coreana*	NC_024548	22,255	complete	Li et al. (2016b)
10		*Rana dabieshanensis*	MW526989	18,291	complete	Unpublished
11		*Rana draytonii*	NC_028296	17,805	complete	Unpublished
24		*Rana dybowskii*	NC_023528	18,864	complete	Li et al. (2016a)
12		*Rana hanluica*	MZ680528	19,395	complete	This study
14		*Rana huanrensis*	NC_028521	19,253	complete	Dong et al., 2016
13		*Rana kukunoris*	KU310893	15,182	partial	Unpublished
15		*Rana kukunoris*	MN733918	16,644	complete	Wang et al., 2020
16		*Rana kukunoris*	NC_035804	18,863	complete	Unpublished
17		*Rana longicrus*	MZ680529	17,833	complete	This study
18		*Rana omeimontis*	MK483118	20,120	complete	Jiang et al., 2020
19		*Rana omeimontis*	NC_035805	19,934	complete	Unpublished
20		*Rana pyrenaica*	KU720300	17,211	complete	Peso-Fernández et al., 2016
21		*Rana temporaria*	NC_042226	16,061	complete	Chen et al., 2018
22		*Rana uenoi*	NC_056272	17,370	complete	Suk et al., 2021
23		*Rana zhenhaiensis*	MN218687	19,205	complete	Huang et al., 2019

Table S2. The mitogenome characteristics and location of the two *Rana* species.

Feature	Strand	*R. hanluica*			*R. longicrus*		
		Position	Intergenic nucleotides	Start/Stop codon	Position	Intergenic nucleotides	Start/Stop codon
trnL(tag)	+	1-72	0		1-72	0	
trnT(tgt)	+	75-144	2		75-144	2	
trnP(tgg)	-	145-213	0		145-213	0	
trnF(gaa)	+	216-285	2		215-284	1	
12S rRNA	+	286-1217	0		285-1215	0	
trnV(tac)	+	1217-1285	-1		1216-1284	0	
16S rRNA	+	1286-2862	0		1285-2862	0	
trnL(taa)	+	2862-2934	-1		2862-2934	-1	
ND1	+	2935-3895	0	GTG/T(AA)	2935-3895	0	GTG/T(AA)
trnI(gat)	+	3896-3966	0		3896-3966	0	
trnQ(ttg)	-	3968-4038	1		3967-4037	0	
trnM(cat)	+	4038-4106	-1		4037-4105	-1	
ND2	+	4107-5141	0	ATG/TAG	4106-5140	0	ATG/TAG
trnW(tca)	+	5140-5209	-2		5139-5208	-2	
trnA(tgc)	-	5210-5279	0		5209-5278	0	
trnN(gtt)	-	5280-5352	0		5279-5351	0	
trnC(gca)	-	5379-5443	0		5379-5443	0	
trnY(gta)	-	5444-5510	0		5444-5510	0	
COX1	+	5512-7065	1	GTG/AGG	5512-7065	1	GTG/AGG
trnS(tga)	-	7057-7127	-9		7057-7127	-9	
trnD(gtc)	+	7129-7197	1		7129-7197	1	
COX2	+	7198-7885	0	ATG/T(AA)	7198-7885	0	ATG/T(AA)
trnK(ttt)	+	7886-7954	0		7886-7954	0	
ATP8	+	7956-8117	1	ATG/TAA	7956-8117	1	ATG/TAA
ATP6	+	8111-8793	-7	ATG/TA(A)	8111-8793	-7	ATG/TA(A)
COX3	+	8793-9577	-1	ATG/TA(A)	8793-9577	-1	ATG/TA(A)
trnG(tcc)	+	9577-9644	-1		9577-9644	-1	
ND3	+	9645-9984	0	ATG/T(AA)	9645-9984	0	ATG/T(AA)
trnR(tcg)	+	9985-10054	0		9985-10054	0	
ND4L	+	10055-10339	0	GTG/TAA	10055-10339	0	GTG/TAA
ND4	+	10333-11692	-7	ATG/T(AA)	10333-11692	-7	ATG/T(AA)
trnH(gtg)	+	11693-11760	0		11693-11760	0	
trnS(gct)	+	11761-11827	0		11761-11827	0	
ND5	+	11860-13647	32	ATG/AGA	11849-13645	21	ATG/AGA
ND6	-	14084-14578	436	ATG/AGA	14316-14810	670	ATG/AGG
trnE(ttc)	-	14579-14647	0		14811-14879	0	
CYTB	+	14651-15793	3	ATG/TAA	14883-16025	3	ATG/TAA
Control region	+	15794-19395	0		16026-17833	0	

Table S3. RSCU information for the mitochondrial protein-coding genes of *R . hanluica* and *R. longicrus*.

Codon	*R. hanluica*		*R. longicrus*		Codon	*R. hanluica*		*R. longicrus*		Codon	*R. hanluica*		*R. longicrus*		Codon	*R. hanluica*		*R. longicrus*	
	Count	RSCU	Count	RSCU		Count	RSCU	Count	RSCU		Count	RSCU	Count	RSCU		Count	RSCU	Count	RSCU
UUU(F)	119	0.91	121	0.94	UCU(S)	61	1.36	63	1.39	UAU(Y)	52	0.9	52	0.89	UGU(C)	8	0.53	10	0.65
UUC(F)	142	1.09	137	1.06	UCC(S)	81	1.81	78	1.73	UAC(Y)	64	1.1	65	1.11	UGC(C)	22	1.47	21	1.35
UUA(L)	106	1	112	1.07	UCA(S)	62	1.38	67	1.48	UAA(*)	3	1.71	3	1.71	UGA(W)	79	1.46	87	1.61
UUG(L)	39	0.37	29	0.28	UCG(S)	10	0.22	7	0.15	UAG(*)	1	0.57	1	0.57	UGG(W)	29	0.54	21	0.39
CUU(L)	112	1.06	110	1.05	CCU(P)	45	0.88	43	0.83	CAU(H)	23	0.44	24	0.45	CGU(R)	10	0.54	8	0.44
CUC(L)	183	1.73	175	1.67	CCC(P)	95	1.86	101	1.96	CAC(H)	81	1.56	83	1.55	CGC(R)	23	1.24	24	1.32
CUA(L)	144	1.36	146	1.39	CCA(P)	51	1	52	1.01	CAA(Q)	67	1.63	68	1.68	CGA(R)	34	1.84	36	1.97
CUG(L)	50	0.47	58	0.55	CCG(P)	13	0.25	10	0.19	CAG(Q)	15	0.37	13	0.32	CGG(R)	7	0.38	5	0.27
AUU(I)	123	0.84	130	0.9	ACU(T)	63	0.88	64	0.87	AAU(N)	51	0.77	54	0.83	AGU(S)	19	0.42	16	0.35
AUC(I)	171	1.16	160	1.1	ACC(T)	112	1.57	110	1.49	AAC(N)	81	1.23	76	1.17	AGC(S)	36	0.8	40	0.89
AUA(M)	106	1.35	110	1.37	ACA(T)	95	1.33	105	1.42	AAA(K)	72	1.73	70	1.71	AGA(*)	2	1.14	1	0.57
AUG(M)	51	0.65	51	0.63	ACG(T)	16	0.22	16	0.22	AAG(K)	11	0.27	12	0.29	AGG(*)	1	0.57	2	1.14
GUU(V)	51	1.02	50	1	GCU(A)	95	1.14	87	1.05	GAU(D)	20	0.56	24	0.68	GGU(G)	28	0.5	24	0.42
GUC(V)	60	1.2	63	1.26	GCC(A)	154	1.84	157	1.9	GAC(D)	52	1.44	47	1.32	GGC(G)	86	1.53	90	1.59
GUA(V)	62	1.24	52	1.04	GCA(A)	71	0.85	71	0.86	GAA(E)	64	1.51	63	1.47	GGA(G)	64	1.14	59	1.04
GUG(V)	27	0.54	35	0.7	GCG(A)	14	0.17	15	0.18	GAG(E)	21	0.49	23	0.53	GGG(G)	47	0.84	53	0.94
